# How reference networks develop, implement, and monitor guidelines

**DOI:** 10.1186/1750-1172-7-S2-A14

**Published:** 2012-11-22

**Authors:** Jan Kirschner, Sunil Rodger, Julia Vry, Kathrin Gramsch, Hanns Lochmüller, Kate Bushby

**Affiliations:** 1 Division of Pediatric Neurology and Muscle Disorders, University Medical Center Freiburg, 79106 Freiburg, Germany; 2 TREAT-NMD, Institute of Genetic Medicine, Newcastle University, International Centre for Life, Central Parkway, Newcastle upon Tyne, NE1 3BZ, United Kingdom

## 

Rare diseases pose many challenges. A paucity of randomised controlled trials for most conditions means that best-practice care guidelines are often non-existent or poorly developed. Where they exist, healthcare professionals may be unaware of them. Furthermore, evaluation of guidelines is difficult, as traditional methods of health-care research (such as hospital admissions and mortality statistics with ICD codes) are not applicable to rare diseases. The CARE-NMD project to improve care for Duchenne muscular dystrophy (DMD) provides an example of how Reference Networks may develop, implement and monitor rare disease guidelines.

The development of the DMD care guidelines was facilitated by the US Centres for Disease Control, and led by patient organisations, translational research networks, and health agencies. In the absence of overwhelming clinical trial evidence, 84 international experts used the RAND/UCLA Appropriateness Method (RAM) to generate consensus on the necessity and appropriateness of clinical interventions and assessments. Yet despite the guidelines, many DMD patients do not receive the treatment they describe.

CARE-NMD has established a Reference Network of care centres for DMD in Europe, to disseminate guidelines, evaluate current practice and identify reasons for non-compliance, and assess guideline impact on quality of life.

Dissemination, via professional and patient networks, has addressed the problem of a lack of awareness of guidelines for this rare disease. Strategies have included presentations at meetings, journal and website publications, media interviews, and professional training courses tailored to local needs in East European partner countries. The ‘Family Guide’, a more accessible version of the care guidelines, is now available in over 20 languages and has been very well received.

To monitor implementation, the project has conducted the largest ever cross-sectional study of DMD patients and their families (n=1677, response rate 66%) across 7 European countries. This assessed – via process and outcome indicators – whether the care they receive aligns with the consensus guidelines, and reported quality of life. In addition, a survey of healthcare professionals has been distributed to care sites in these countries via the Care and Trial Site Registry (CTSR), an online self-registration platform for neuromuscular centres developed by the TREAT-NMD network of excellence. This now includes information on patient cohort, care settings, research activities and clinical trial capabilities of more than 200 sites.

A Reference Network such as CARE-NMD enables implementation, assessment and monitoring of care guidelines for rare diseases. The data it gathers will be crucial in further developing and refining guidelines.

